# Design and Simulated Electrical Properties of a Proposed Implanted-Epi Silicon 3D-Spherical Electrode Detector

**DOI:** 10.3390/mi14030551

**Published:** 2023-02-26

**Authors:** Xinyi Cai, Zheng Li, Xinqing Li, Zewen Tan, Manwen Liu, Hongfei Wang

**Affiliations:** 1 College of Physics and Optoelectronic Engineering, Ludong University, Yantai 264025, China; 2 College of Integrated Circuits, Ludong University, Yantai 264025, China; 3 Engineering Research Center of Photodetector Special Chip in Universities of Shandong, Ludong University, Yantai 264025, China; 4 School for Optoelectronic Engineering, Zaozhuang University, Zaozhuang 277160, China; 5 Institute of Microelectronics, Chinese Academy of Sciences, Beijing 100029, China; 6 School of Physics and Physical Engineering, Qufu Normal University, Qufu 273165, China

**Keywords:** Implanted-Epi Silicon 3D-Spherical Electrode Detector, electrical characteristic, full three-dimensional simulation, full depletion voltage, detector array, photon science

## Abstract

A new type of 3D electrode detector, named here as the Implanted-Epi Silicon 3D-Spherical Electrode Detector, is proposed in this work. Epitaxial and ion implantation processes can be used in this new detector, allowing bowl-shaped electrodes to penetrate the silicon completely. The distance between the bowl cathode and the central collection electrode is basically the same, thus the total depletion voltage of Implanted-Epi Silicon 3D-Spherical Electrode Detectors is no longer directively correlated with the thickness of the silicon wafer, but only related to the electrode spacing. In this work, we model the device physics of this new structure and use a simulation program to conduct a systematic 3D simulation of its electrical characteristics, including electric potential and electric field distributions, electron concentration profile, leakage current, and capacitance, and compare it to the traditional 3D detectors. The theoretical and simulation study found that the internal electric potential of the new detector was smooth and no potential saddle point was found. The electric field is also uniform, and there is no zero field and a low electric field area. Compared with the traditional silicon 3D electrode detectors, the full depletion voltage is greatly reduced and the charge collection efficiency is improved. As a large electrode spacing (up to 500 μm) can be realized in the Implanted-Epi Silicon 3D-Spherical Electrode Detector thanks to their advantage of a greatly reduced full depletion voltage, detectors with large pixel cells (and thus small dead volume) can be developed for applications in photon science (X-ray, among others).

## 1. Introduction

A semiconductor detector is a kind of solid radiation detector; the most commonly used semiconductor materials are silicon and germanium [1,2]. Silicon detectors [3] are radiation detectors [4] with silicon as the detection medium, which have the characteristics of high sensitivity, small size, and easy integration. Thanks to their strong practicability and mature technology, they have gradually developed into the most mature radiation detector [5], and have been widely used in medical [6], security, aerospace [7], and high energy physics experiments [8,9,10]. In 1997, Parker et al. of the University of Hawaii proposed the first 3D detector, a 3D-column electrode silicon detector [11]. Compared with the previous two-dimensional detector, the cylindrical electrode of this detector extends to the silicon substrate [12,13,14,15]. There is a symmetry point between two p-type electrodes and two n-type electrodes, where the value of the electric field is zero [16,17,18,19]. To solve this problem, in 2009, Brookhaven National Laboratory proposed a new type of 3D detector, a 3D-Trench electrode detector [20]. The 3D-Trench electrode detector has two major advantages over the conventional 3D column electrode detector: (1) there is nearly no angle dependence in electric field and charge collection and (2) if the junction is located at the trench electrode, the electric field is much lower and more uniform than that of 3D column electrode detectors [21,22,23]. 

With the continuous progress of the process in recent years, especially the achievements in the nanometer order of magnitude, the maturity and stability of etching have been improved [24,25,26]. This brings great convenience to the production of traditional 3D electrode detectors. However, traditional 3D electrode detectors and 3D-Trench electrode detectors need to be deeply etched [27], which will greatly damage the mechanical stability of the detector. In order to improve the mechanical stability and prevent the electrode from falling off, an unetched substrate is left at the bottom of the 3D-Trench electrode detector [28]. However, even if the thickness of this unetched substrate in the 3D-Trench electrode detector is limited to 50 μm, there is still about 10% of the unetched volume [29]. When the detectors are arrayed, the electrical signal between each detector cell will affect other cells through the substrate, resulting in a low energy resolution of the detector [30]. Moreover, the values of the electric field in the unetched volume are very low or even close to zero, forming a ‘dead space‘. The existence of the dead zone means the electrons in this region are unable to drift to the electrode in time because of the low electric field, which is very unfavorable to the performance of the detector. Therefore, it is necessary to develop a new type of detector without dead space. 

**Figure 1:**
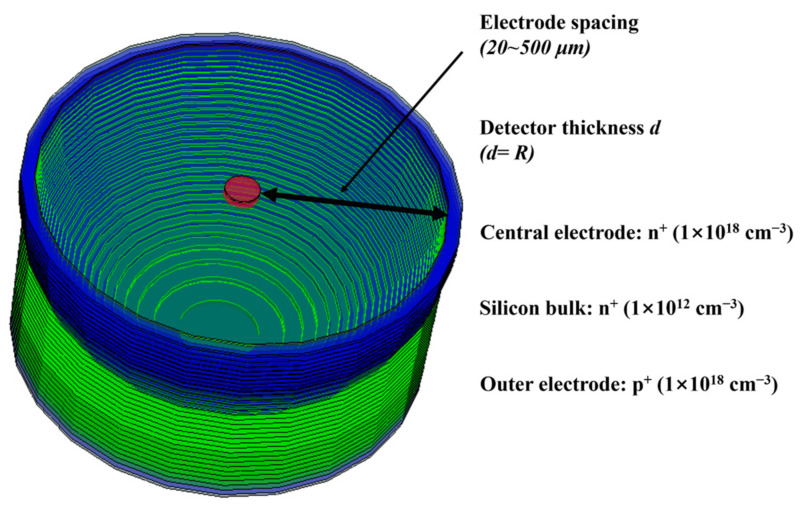
A single cell of the Implanted-Epi Silicon 3D-Spherical Electrode Detector. The effective doping density can be reasonably adjusted according to the actual situation.

In the previous work, we assumed and analyzed a hemispherical detector model, but this detector cannot be read or manufactured [31]. Then, we provided a scheme for manufacturing a detector, but this scheme still leaves a substrate in the detector [32]. In this work, we propose a new type of 3D electrode detector, an Implanted-Epi Silicon 3D-Spherical Electrode Detector. As shown in Figure 1 for a single-cell of the Implanted-Epi Silicon 3D-Spherical Electrode Detector, it can be seen that the 3D-bowl cathode of the detector penetrates the whole silicon body and uniformly covers the anode. In this work, we model the device physics and simulate this new detector to explore its simulated electrical properties.

## 2. The Concept and Design of Implanted-Epi Silicon 3D-Spherical Electrode Detectors

According to recent research progress, the research of new detector materials for photoelectric and ionizing radiation detection has brought us inspiring results. They have great potential with a high signal-to-noise ratio and fast response [33,34,35]. As a mature semiconductor detector material, silicon has a better guarantee in process maturity and operation stability. Therefore, we continue to use silicon as the substrate of the detector. The basic shapes of the detector unit cell can be hexagon, square, or circular. The array composed of square elements is the simplest, but the distance between the anode and cathode of the detector unit cell is uneven in this arrangement. The hexagon shape is the closest to the circle, which makes it an effective array. To study the device physics of a single cell, without losing generality, we choose the circular shape in our study.

**Figure 2:**
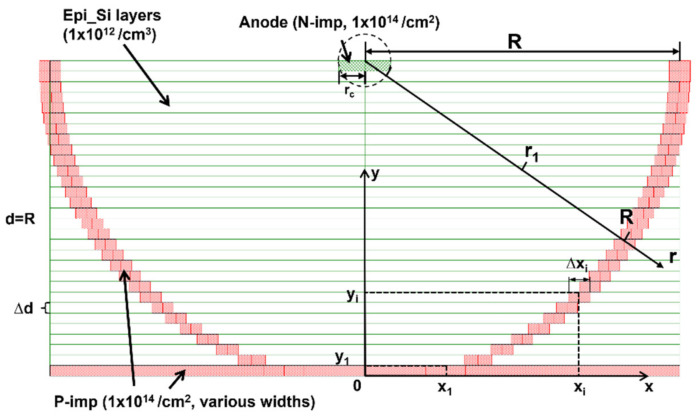
The section rendered image of the Implanted-Epi Silicon 3D-Spherical Electrode Detector unit cell center.

As shown in Figure 2, we designed a new type of bowl-shaped (semi-sphere-shaped to be exact) electrode to achieve uniform spacing between the anode and cathode. The central collection electrode is at the center of the top surface of the detector, which is the anode of the detector, and we set the contact of the cathode on the bottom of the detector. In the detector single cell, the anode is uniformly surrounded by a bowl cathode with a uniform electrode spacing.

The coordinate system is established with the bottom center of the detector unit cell as the origin. The thickness of each layer of the bowl-electrode slice is Δd, and the number of layers is N=R/Δd. We define the point at which the coordinate origin is located as the center of the bottom of the first layer. Then, the ordinate at the top of the center of the *i*-th layer bowl-electrode is $y_{i}=i\Delta d\left(i=1,\ldots ,N\right)$, and the abscissa is $x_{i}=\sqrt{R^{2}-{\left(R-i\Delta d\right)}^{2}}$. Both $x_{i}$ and $y_{i}$ are functions of i, and the differential of $x_{i}$ can be obtained:

(1) $$dx_{i}=d\left(\sqrt{R^{2}-{\left(R-i\Delta d\right)}^{2}}\right)=\frac{\Delta d\left(R-i\Delta d\right)di}{\sqrt{R^{2}-{\left(R-i\Delta d\right)}^{2}}}$$

For finite increments, we can replace d with delta (use symbol here), thus the formula for the cathode width of the ion implanted (p^+^ implantation here) on the *i*-th layer can be obtained:

(2) $$\Delta x_{i}=\frac{\Delta d\Delta i\left(R-i\Delta d\right)}{\sqrt{R^{2}-{\left(R-i\Delta d\right)}^{2}}}=\frac{\Delta d\left(R-i\Delta d\right)}{\sqrt{R^{2}-{\left(R-i\Delta d\right)}^{2}}}=\Delta d\frac{\left(1-i\frac{\Delta d}{R}\right)}{\sqrt{2i\frac{\Delta d}{R}-{\left(i\frac{\Delta d}{R}\right)}^{2}}}$$

Because $\Delta x_{N}=0$, we can let $\Delta x_{N}=\Delta x_{N-1}$. If $\Delta x_{N-1}$ is too small, we can let $\Delta x_{N-1}=\Delta x_{N-2}$, and so on, until we find a large enough $\Delta x_{N-m}$. $\Delta x_{N-m}$ depends on the minimum width of ion implantation. In this work, we set $\Delta x_{N-m}$ to 4 μm.

**Figure 3:**
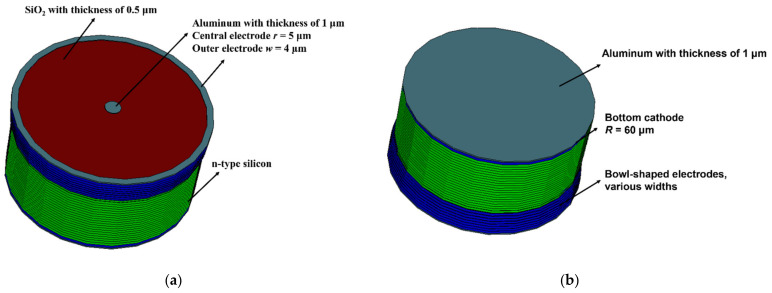
The view of the Implanted-Epi Silicon 3D-Spherical Electrode Detector: (**a**) top view and (**b**) bottom view. The outer surface of the electrode is covered with 1 μm thick aluminum to connect to the circuit.

In this work, we choose 60 μm as the radius of the detector, and the radius of the detector anode is 5 μm. Then, the position coordinates of the bowl electrode can be calculated from the above formula. Δd is 2 μm and the minimum width of the bowl electrode is set to 4 μm. The new detector can have four doping methods, as shown in Table 1. We adopted the n^+^/n/p^+^ type, and each Epi Silicon layer is N doped with a concentration of 1 × 10^12^ cm^−3^, which is called the effective doping concentration *N_eff_*. The anode of the detector is N^+^ doped with a concentration of 1 × 10^18^ cm^−3^, while the ion implanted 3D-bowl cathode and bottom cathode are P^+^ doped with a concentration of 1 × 10^18^ cm^−3^, thus forming the PN junction. The junction near the bowl electrode can ensure good electric field distribution and low full depletion voltage. At the top of the detector, a 1 μm aluminum layer is used to just cover the central anode, while a 0.5 μm SiO_2_ layer is used to cover elsewhere. At the bottom of the detector, the entire ion implant area is covered with a 1 μm aluminum layer. Figure 3 shows the top and bottom of the Implanted-Epi Silicon 3D-Spherical Electrode Detector.

Based on the current process level, it is difficult to directly manufacture the 3D spherical detector. Here, we provide a preferred process in actual production—that is, the epitaxial process combined with the ion implantation process. This scheme enables electrodes to penetrate the entire silicon substrate and uses two-dimensional technology to achieve the purpose of manufacturing three-dimensional detectors. The epitaxial process [36] can be combined with the ion implantation process [37] in actual production. Taking a detector with a radius of 60 μm as an example, the silicon is composed of 30 layers of silicon wafers with a thickness of 2 μm epitaxial growth. Each layer of the thin silicon wafer is grown by the epitaxial process and then doped by ion implantation on the set position. After the silicon body part is made, silicon wafer oxidation and aluminum deposition are carried out. This layered ion implantation method makes the transverse diffusion of impurities very small when the anode and cathode are doped, which improves the controllability of the internal structure of the detector. At the same time, the matching degree of silicon between each layer of the detector and the bonding strength of epitaxial growth are both high. The ion implantation process also improves the selection range of doped impurities, which can be a number of different types of ions. It is also much simpler and cleaner than the ion diffusion method. This will provide a preferred practical process plan for future laboratory tape-out. In this work, we will use simulation software to test its electrical properties.

This structure entirely avoids etching of trenches in the Si bulk, which not only eliminates the shortcomings of dead space in 3D trench electrode detectors [21,29], but also provides a solid, robust detector structure without hollow trenches or trench fills in the detector bulk. It is also a detector with entire planar technology and ion implantation, compatible with current Si processing technology. Moreover, it is a detector with a complete electric shielding of the central collection electrode by the spherical electrode, making it possible to realize a true pixel detector without the interference of neighboring pixels.

The structure of the detector is symmetrical, and we can have two different detector configurations for the same matrix doping type detector: (1) the junction is at the outer ring spherical shell electrode, named as 3D-sphere-ORJ; and (2) the junction is at the center dot electrode, named as 3D-sphere-CJ. The depletion voltage of 3D-sphere-ORJ is lower than that of 3D-sphere-CJ [21]. This means that the voltage of the detector is lower when it works normally, which saves energy and means it is not broken down easily. The electric potential and field will be vastly different for the two cases; 3D-sphere-ORJ is selected here.

By solving the Poisson equations,

(3) $$\frac{1}{r^{2}}\frac{d\left(\frac{r^{2}d\varphi \left(r\right)}{dr}\right)}{dr}=-\frac{eN_{eff}}{\varepsilon \varepsilon_{0}}$$

With the following boundary conditions:

(4) $$\left\{\begin{array}{c} \varphi \left(R\right)=-\left|V\right|\\ \varphi \left(r_{1}\right)=0\\ E\left(r_{1}\right)=0 \end{array}\right.$$

We can obtain the electric potential in a single cell:

(5) $$\varphi \left(r\right)=\frac{eN_{eff}}{6\varepsilon \varepsilon_{0}}\left(R^{2}-r^{2}\right)+\frac{eN_{eff}r_{1}{}^{3}}{3\varepsilon \varepsilon_{0}}\left(\frac{1}{R}-\frac{1}{r}\right)-\left|V\right|$$

when $r=r_{1}=r_{c}$, $\varphi \left(r_{1}\right)=\varphi \left(r_{c}\right)=0$, $\left|V\right|=V_{fd}$

We obtain the full depleted voltage as follows:

(6) $$V_{fd}=\frac{eN_{eff}}{6\varepsilon \varepsilon_{0}}\left(R^{2}-r_{c}{}^{2}\right)+\frac{eN_{eff}r_{c}{}^{3}}{3\varepsilon \varepsilon_{0}}\left(\frac{1}{R}-\frac{1}{r_{c}}\right)$$

and the electric field strength in a single cell

(7) $$E\left(r\right)=\frac{eN_{eff}}{3\varepsilon \varepsilon_{0}r^{2}}\left(r^{3}-r_{1}{}^{3}\right)$$

where $\varphi \left(r_{1}\right)$ is the potential at the edge of the PN junction depletion layer in the detector unit, $\varphi \left(r\right)$ is the potential of any point r in the detection region, $\varphi \left(R\right)$ is the potential at the outer edge of the bowl cathode, $\left|V\right|$ is the absolute value of the applied voltage, $r_{1}$ is the distance between the edge of the depletion region and the anode center, R is the distance between the anode center and the bowl electrode, e is the basic charge, $N_{eff}$ is the effective doping concentration of the n-type bulk, $r_{c}$ is the anode radius, $\varphi \left(r_{c}\right)$ is the potential of the anode, ε is the relative dielectric constant of silicon with a value of 11.9, and $\varepsilon_{0}$ is the vacuum dielectric constant with a value of 8.854 × 10^−12^ F/m.

In the process of simulation, we define the carrier mobility in the physical model, which includes carrier–carrier scattering, doping-dependent mobility, high-field saturation models (e high field saturation and h high field saturation) under gradient of quasi-Fermi potential, and mobility degradation at interfaces. In addition, the oxide charge density is 4 × 10^11^ cm^−2^ for both sides. We also set the Shockley–Read–Hall (SRH) doping dependence and Van Dort model [38]. The bandgap model (effective intrinsic density) activates the bandgap narrowing effect of silicon in the highly doped region, which directly affects the calculation of the intrinsic carrier density in silicon. At the same time, the electron hole Poisson is added to the couple.

## 3. Electrical Characteristic Results

### 3.1. Electrical Potential Distribution

**Figure 4:**
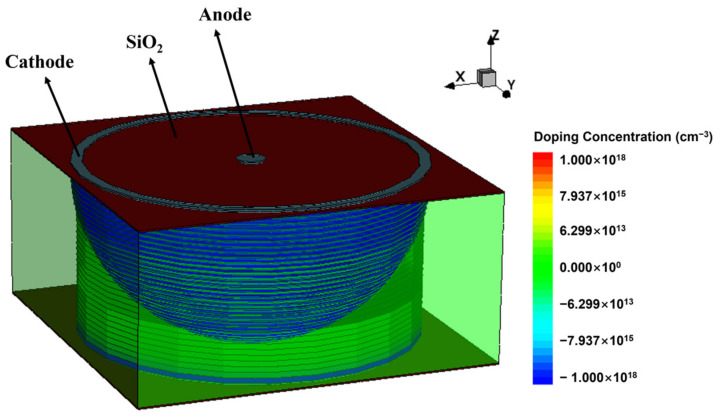
The full three-dimensional simulation structure of the Implanted-Epi Silicon 3D-Spherical Electrode Detector. The doping concentration of cuboids outside the detector unit cell is equivalent to that of silicon substrate.

In this work, we used the TCAD tool to simulate the Implanted-Epi Silicon 3D-Spherical Electrode Detector unit cell and studied its electrical characteristics. Figure 4 identifies the electrode structure and doping concentration in the detector unit cell. We applied different negative voltages on the cathode electrode and zero bias voltage on the anode electrode to obtain the electrical characteristics of the new detector structure under the condition of different bias voltages without irradiation.

**Figure 5:**
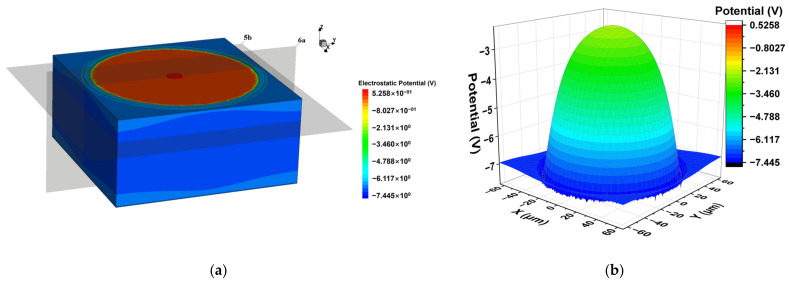
Electric potential distribution of the detector at −7 V bias voltage: (**a**) 3D distribution; (**b**) the 2D section diagram at cutting plane z = 45 μm. The two gray surfaces in (**a**) are the sections of Figure 5b and Figure 6a.

The electric potential, electric field, and electron distribution are significant characteristics of the Implanted-Epi Silicon 3D-Spherical Electrode Detector and we conducted a full 3D simulation. For the purpose of better viewing, we construct 2D cutting planes (e.g., as shown in Figure 5a) of the detector and display these distributions. Shown in Figure 5b is a 3D plot of the electric potential on the cutting plane at z = 45 μm (as shown in Figure 5a). The electric potential forms a mountain-like shape with a cylindrical symmetry in the sensitive area of the detector, with the value near the center being the highest and decreasing from the center to the surroundings. The electric potential gradient (the electric field) is smooth and electrons will drift along the potential gradient towards the center region under the anode for collection.

**Figure 6:**
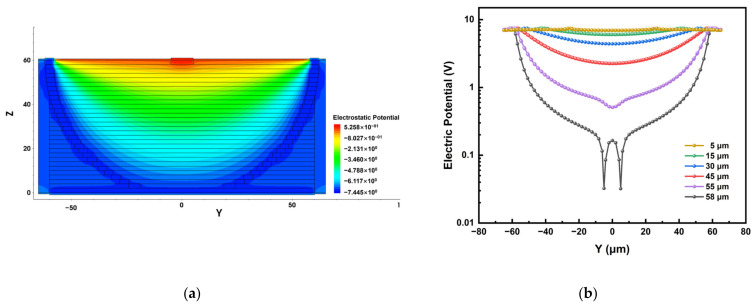
Potential distribution of the detector: (**a**) cutting plane z = 57 μm; (**b**) linear diagram of different y-axis heights. For convenience, we use the absolute value to display these data. The broken line at 58 μm in (**b**) is caused by the collection electrode (the cutting line passes through the anode).

In order to further analyze the electric potential distribution of the detector, we cut the central collection electrode of the detector horizontally and vertically to obtain the plane electric potential distributions, as shown in Figure 6. We can clearly see that the electric potential in the sensitive region of the detector presents a uniform and symmetrical distribution, and no electric potential saddle point is found in the sensitive region of the detector, which will be good for charge collection.

### 3.2. Electric Field Distribution

**Figure 7:**
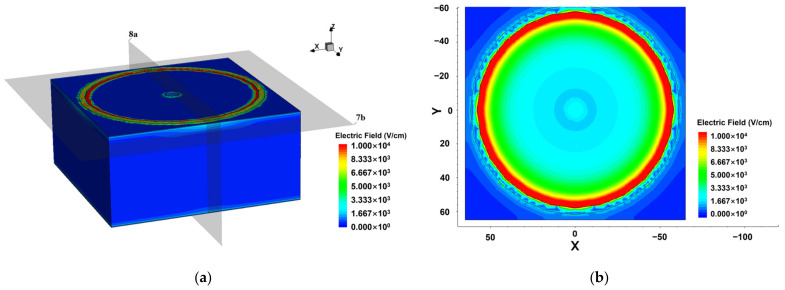
Electric field distribution of the detector at −7 V bias voltage: (**a**) 3D distribution; (**b**) the 2D section diagram at cutting plane z = 57 μm. The two gray surfaces in (**a**) are the sections of Figure 7b and Figure 8a.

Another important characteristic of the Implanted-Epi Silicon 3D-Spherical Electrode Detector is the electric field distribution. In order to clarify the electric field distribution inside the detector, we construct the cutting plane of the structure at z = 57 μm to better demonstrate the electric field and potential distributions. As shown in Figure 7, it can be seen that the surface electric field strength of the detector is low, and the detector is not susceptible to surface breakdown because the substrate is an n-type semiconductor material, which does not form a PN junction on the surface. While the electric field distribution at the cross section shows a concentric ring shape, which decreases from the outer cathode to the inner central collecting electrode, this uniform electric field will help the detector to collect the charge. The distance of the detector unit cell from the cathode to the central anode is the same at all angles. Therefore, the electric field distribution has almost no angle dependence, only varying with r in a spherical coordinate.

**Figure 8:**
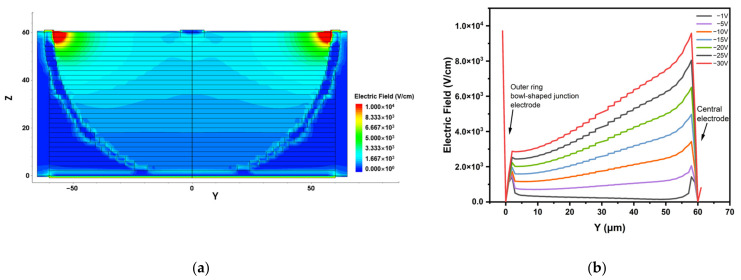
The electric field distribution of the detector: (**a**) the 2D section diagram at cutting plane x = 0 μm; (**b**) 1D electric field profiles at various bias voltages. The black line segment in (**a**) is the cutting surface in (**b**).

In order to further examine the electric field distribution inside the detector, we view the two-dimensional electric field distribution along the x-axis of the detector, as shown in Figure 8a. It can be seen that the highest electric field inside the detector appears near the cathode (gradually decreasing from cathode to anode), and its maximum value is confirmed to be about 3.7 × 10^4^ V/cm. There is no dead space in the sensitive region of the detector, which will greatly improve the charge collection of the detector. The one-dimensional linear electric field strength under different bias voltages is also extracted, as shown in Figure 8b. With the increase in bias voltage, the electric field in the sensitive region of the detector increases continuously until it reaches the carrier saturation drift velocity.

The whole detector sensitive region has a non-zero electric field. Carriers will move fast with high drift velocity, and the probability of trapping by deep level defects will be reduced.

### 3.3. Electronic Concentration Distribution and Depletion Voltage

**Figure 9:**
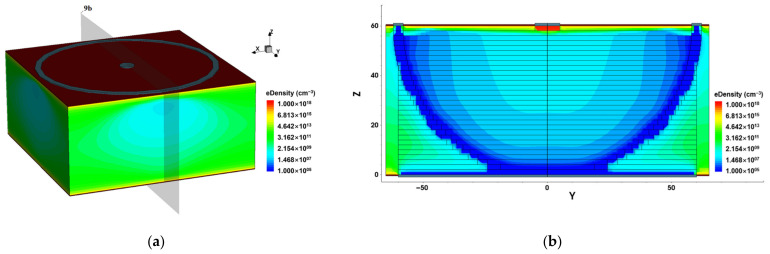
Electronic concentration distribution of the detector at −7 V bias voltage: (**a**) 3D distribution; (**b**) the 2D section diagram at cutting plane x = 0 μm. The gray plane in (**a**) is the cutting surface in (**b**).

Figure 9 shows the electron concentration distribution of the detector, where the electron concentration is low near the inner side of the bowl-electrode and becomes higher closer to the central collection electrode. Combined with the electric potential and electric field distribution of the detector, it is found that the potential in the middle of the detector is higher than that near the bowl cathode. Thus, electrons will drift towards the center anode while holes will drift in the opposite direction to the bowl-electrode (cathode).

Full depletion voltage is the minimum bias voltage needed to completely deplete the volume between the cathode and anode electrodes. After the detector is applied with a bias voltage, the depletion region increases with the increase in applied voltage until it reaches the full depletion. In order to obtain the maximum sensitive area and achieve the highest detection efficiency, the detector usually needs to work under the condition of full depletion. We obtain the exact value of the full depletion voltage by analyzing the electron concentration under different bias voltages.

**Figure 10:**
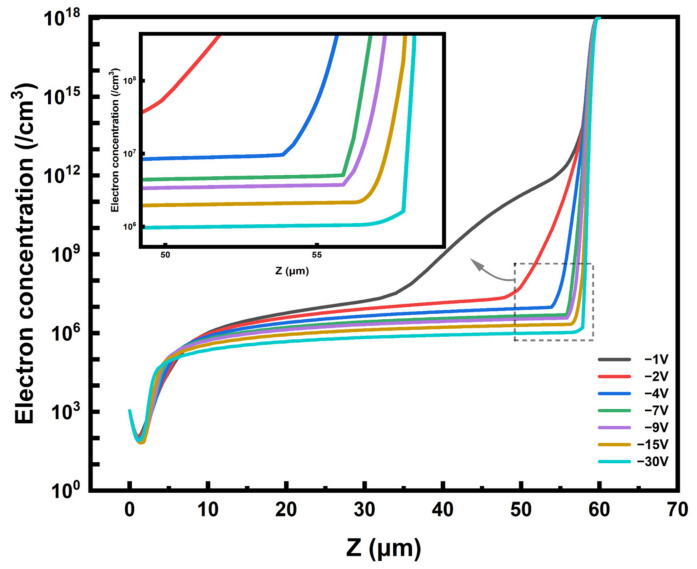
The electron concentration distribution at different bias voltages. The black line segments in Figure 9b are the cut line surfaces.

In Figure 10, we extract the one-dimensional electron concentration curve on the straight line of the detector unit z-axis (x = 0, y = 0). With the increase in bias voltage, the electron concentration decreases continuously (the sensitive region increases continuously) until it is completely depleted. It can be observed that the electron concentration in much of the detector is basically lower than that of the substrate doping concentration (1 × 10^12^ cm^−3^) at a bias voltage of −2 V. The deep depletion occurs at −7 V, and the electron concentration in the depletion region does not change significantly with the increase in bias voltage. In general, when the electron concentration in the sensitive region of the detector is basically lower than the effective doping concentration of the silicon substrate, the detector is considered to be exhausted. Therefore, we determine that the full depletion voltage of the new detector is −7 V, which is much lower than that of conventional detectors [28,39].

### 3.4. Leakage Current and Capacitance

**Figure 11:**
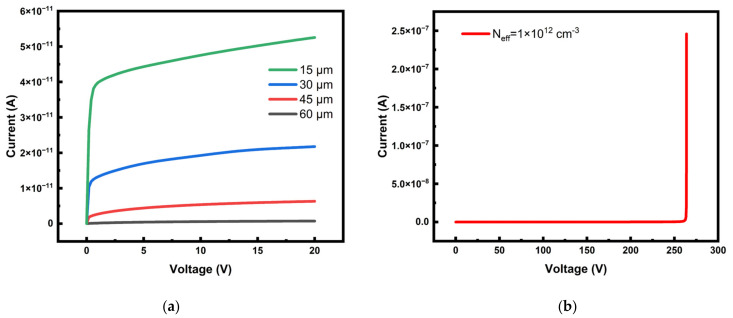
The relationship between leakage current curve and bias voltage. (**a**) Leakage currents with different radii at a bias voltage of −20 V. The detector is reduced in proportion to the radius, so the electrode spacing is also reduced. (**b**) Leakage current when the bias voltage is −300 V and the radius is 60 μm. The abscissa of the point where the leakage current suddenly increases is the junction breakdown voltage.

The detector persists a leakage current after the detector is biased, and its size is affected by the structure (depletion volume) [40]. In our case, the surface charge is set to 4 × 10^11^ cm^−2^, and a bias voltage of −20 V is applied to the detector. It can be observed from Figure 11a that, as the voltage increases, the leakage current gradually increases and tends to be saturated. The bias voltage and leakage current are displayed in their absolute values for easy reading. The leakage current increases with the increase in electrode spacing (small effective detector area), and the detector with small electrode spacing may have better performance in the environment with high radiation fluence [40]. The corresponding carriers are generated by the SRH generation-recombination process, as described by the SRH equation. The leakage current of the detector with 60 μm electrode spacing (radius R) is 55.2 pA. The breakdown voltage is the maximum voltage that can be applied under the working state of the detector. Figure 11b shows the breakdown characteristics of the detector at a radius of 60 μm. The leakage current of the detector increases rapidly after the bias voltage reaches 261 V, and then the detector is broken down. The breakdown voltage of our new detector if much higher than the 174 V value of the 3D-Trench detector [41]. The breakdown voltage is two orders of magnitude larger than the detector full depletion voltage and the detector leakage current after full depletion remains stable before breakdown.

**Figure 12:**
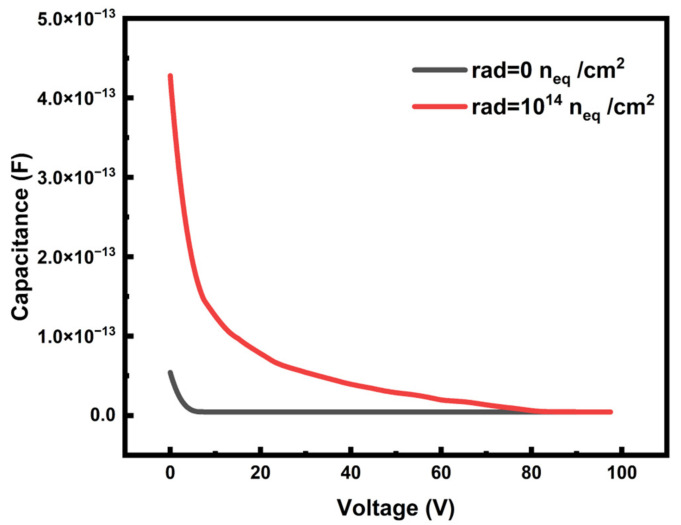
Capacitance in different working environments. The bias voltage is displayed in absolute value. The capacitance tends to be stable after decreasing to 4.48 fF.

As shown in Figure 12, the capacitance of the detector is larger in the radiation environment at a low bias voltage. As the voltage increases, the capacitance continues to decrease until it reaches a saturated capacitance of 4.48 fF, which is extremely small. The capacitance of the 3D-Trench detector is usually 80–100 fF, which is much higher than the capacitance of the new detector [28,42]. The capacitance is only related to the structure of the detector and has nothing to do with the environment of the detector. Therefore, in the irradiation environment, the saturated capacitance remains unchanged.

## 4. Detector Array of the Implanted-Epi Silicon 3D-Spherical Electrode Detector Cells

**Figure 13:**
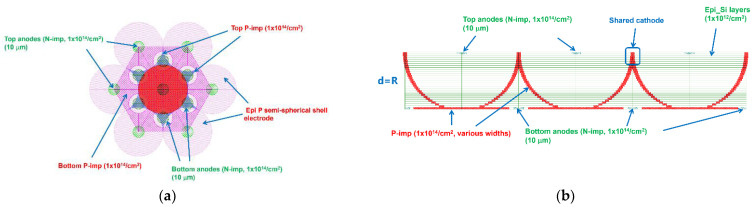
Detector array of the Implanted-Epi Silicon 3D-Spherical Electrode Detector Cells: (**a**) prone view and (**b**) cross-sectional perspective view. In practice, the top anode and the bottom anode in (**b**) do not appear in the same plane, which is to facilitate the display of the internal structure of the detector.

The detector array is composed of detector cells and we add additional anodes to the junction at the bottom of the cells, as shown in Figure 13. The bottom anode eliminates the dead space in the triangular cone-like region of the array, so that there is no dead space in the entire detector array. Furthermore, we added an electrode protection ring (p-type implants) around the bottom anode to prevent shorts between bottom anodes. The adjacent detector cells can share the upper region of the bowl-shaped electrode benefiting from the construction of the new detector. The effective incident area of the detector is expanded because the design of the shared cathode reduces the electrode area. These detector arrays can be used in photon physics experiments and other scientific research fields thanks to their lack of dead space, high position resolution, and high charge collection efficiency properties.

## 5. Conclusions

In this work, a new three-dimensional electrode silicon detector was proposed and described in detail, and its electrical properties were simulated and studied. It also provides a feasible way to fabricate a 3D spherical electrode detector, which is compatible with current Si processing technology and can realize a large detector array to develop a 3D spherical electrode pixel detector. In this new detector, the distance between the detector bowl-electron and the central collection electrode is the same. Therefore, the electric field and charge collection characteristics are almost independent of the angle, and the depletion voltage is only related to the electrode spacing. Compared with the traditional 3D detector, the new detector bowl cathode and anode spacing are the same, the dead space problem is overcome, and the electrical performance is improved. The leakage current and capacitance is weak and the breakdown voltage is much higher than the full depletion voltage. Detector arrays will be studied in further detail in the future.

The simulation results show that the new detector has uniform electric potential distribution without a saddle point, which is of great help to the charge collection. The electric field distribution shows that the electric field in the sensitive area of the detector is smooth and uniform, without a high field region to cause local breakdown. This design has no dead space between the cathode and anode electrodes inside the detector. We use the electron concentration under different bias voltages to determine the depletion voltage of the detector, which is only a few volts, much smaller than 2D and 3D-Trench electrode detectors with the same electrode spacing. The above theoretical and simulation results will provide strong support for the manufacturing and testing of future detectors.

## Figures and Tables

**Table 1:** Doping method of the detector.

Doping Method	Central Electrode	Silicon Bulk	Outer Electrode
p^+^/p/n^+^	p^+^	p	n^+^
p^+^/n/n^+^	p^+^	n	n^+^
n^+^/n/p^+^	n^+^	n	p^+^
n^+^/p/p^+^	n^+^	p	p^+^

## Data Availability

The data presented in this study are available from the corresponding author upon reasonable request.
